# Generalization of intrinsic ductile-to-brittle criteria by Pugh and Pettifor for materials with a cubic crystal structure

**DOI:** 10.1038/s41598-021-83953-z

**Published:** 2021-02-25

**Authors:** O. N. Senkov, D. B. Miracle

**Affiliations:** Air Force Research Laboratory, Materials and Manufacturing Directorate, Wright-Patterson AFB, USA

**Keywords:** Mechanical engineering, Mechanical properties

## Abstract

Two classical criteria, by Pugh and Pettifor, have been widely used by metallurgists to predict whether a material will be brittle or ductile. A phenomenological correlation by Pugh between metal brittleness and its shear modulus to bulk modulus ratio was established more than 60 years ago. Nearly four decades later Pettifor conducted a quantum mechanical analysis of bond hybridization in a series of intermetallics and derived a separate ductility criterion based on the difference between two single-crystal elastic constants, C_12_–C_44_. In this paper, we discover the link between these two criteria and show that they are identical for materials with cubic crystal structures.

## Introduction

Ductility is an essential property, it allows materials to be shaped into useful parts and to accept limited damage in service without failing. Brittle materials cannot be formed into components using conventional methods, and cannot be used as structural materials since failure can occur unexpectedly due to defects and stress concentrations. There are several well-known methods for strengthening materials along with physical models that can accurately predict strength. However, there are no general, physical models for predicting ductility^1^. Therefore, it is important for materials discovery and development to have rules that can predict if a selected composition will be brittle or ductile. In the early 1950′s Pugh used empirical reasoning that linked the shear modulus, G, with ductility and the bulk modulus, B, with fracture. After analyzing experimental data from dozens of elemental metals, Pugh found that metals with a small ratio, G/B, were generally ductile, whereas metals with a high G/B ratio were generally brittle^2^. Pugh did not propose a critical G/B value for the transition from ductile to brittle behavior; however, later analysis of a number of crystalline alloy systems suggested that brittle behavior would be common when G/B ≥ 0.57–0.6^1,3–5^, while in metallic glasses a sharp transition was observed at G/B ≈ 0.41–0.43^6,7^.

In the late 1980s and early 1990s Pettifor^8,9^ conducted a quantum mechanical analysis of bond hybridization in a series of intermetallics and derived a separate ductility criterion based on the difference between two single-crystal elastic constants, C_12_ and C_44_. According to Pettifor’s criterion, which was derived using a many body potential that explicitly includes the angular character of the bonding orbitals^8,10^, material with non-directional metallic bonds is intrinsically ductile and has a positive Cauchy pressure, C” = C_12_ − C_44_ > 0; whereas material with negative Cauchy pressure (C” < 0) possesses directional, covalent bonding and is intrinsically brittle^8,9^. Since then, efforts to predict whether a material will be brittle or ductile have used one or the other of these two (Pugh or Pettifor) criteria.

**Figure 1:**
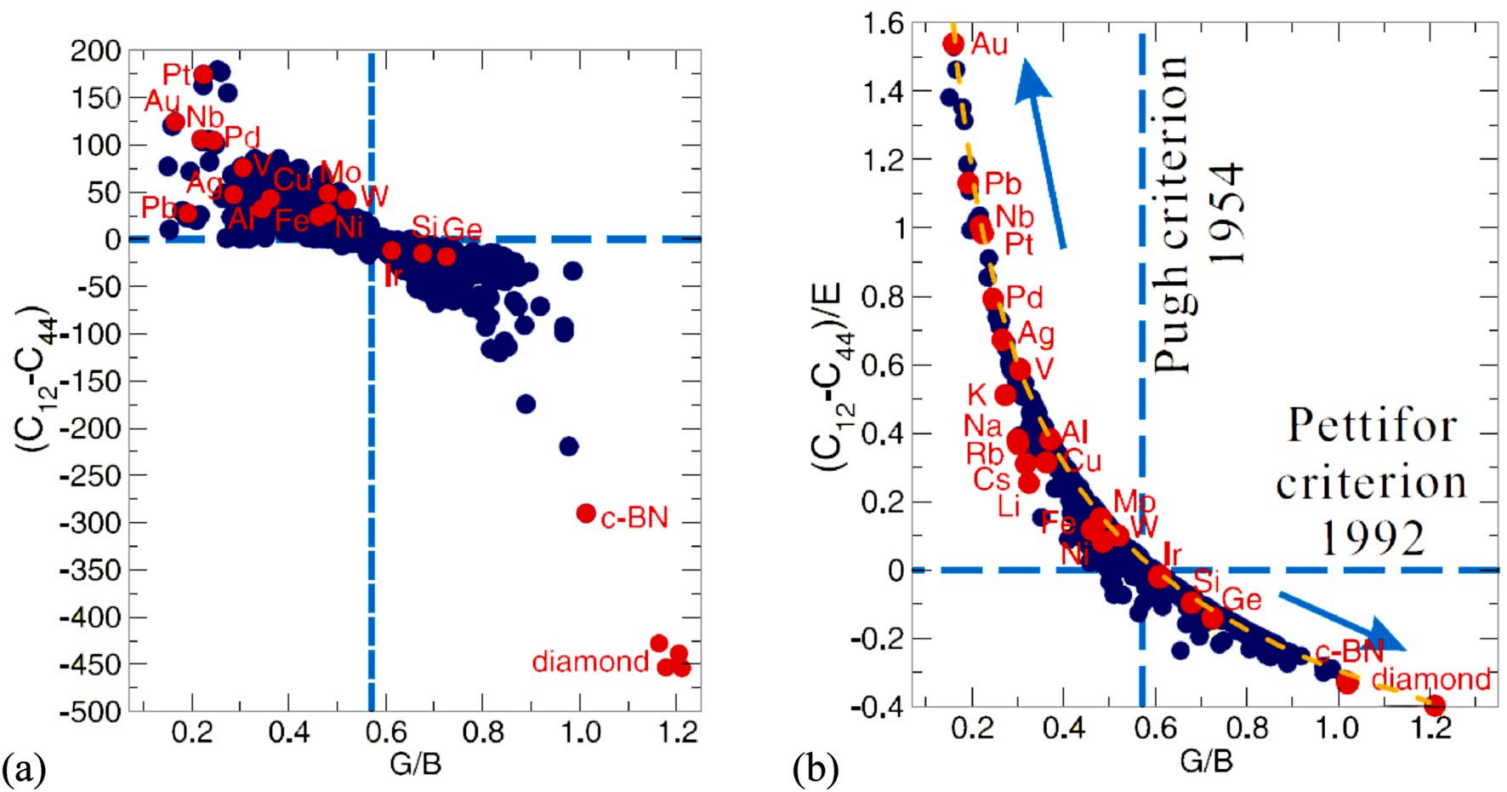
(**a**) Correlation between C” = (C_12_ − C_44_) and G/B for 308 compounds and 24 metals given in Supplementary materials of^5^; (b) a renormalized hyperbolic correlation between C” normalized by Young modulus E and G/B for all the data from (**a**). The horizontal dashed line corresponds to C” = 0 and the vertical dashed line corresponds to G/B = 0.57. (Compiled from^5^.) A dashed yellow data trendline in (**b**) corresponds to Eq. (8).

Recently Niu et al*.*^5^ analyzed the properties of a number of ductile and brittle materials with cubic crystal structures. While previous efforts have generally used either Pugh^2^ or Pettifor^8,9^ criteria to identify the intrinsic ductile-to-brittle transition as a function of elastic properties, Niu et al*.*^5^ attempted to find a correlation between these criteria by analyzing 308 intermetallic compounds and 24 metals and semi-metals. When they plotted C” versus G/B, a very large scatter was found indicating poor correlation between these parameters (Fig. 1a). However, when C” was normalized by Young’s modulus E (*i.e.* when the C”/E ratio was used), the materials followed a broadly hyperbolic trend (Fig. 1b). Unfortunately, an explanation of the origin of this intriguing correlation was not given.

In the present report, we derive a relationship between the Cauchy pressure and Pugh’s modulus ratio and show that these two classical criteria are identical for materials with cubic crystal structures.

## Results and discussion

For cubic crystals, the macroscopic Voigt shear modulus G_v_ and bulk modulus B can be expressed as functions of the elastic constants C_11_, C_12_ and C_44_^11,12^:

1 $$ {\text{5G}}_{{\text{v}}} = {\text{3C}}_{{{44}}} + {\text{C}}_{{{11}}} - {\text{C}}_{{{12}}} $$

2 $$ {\text{3B}} = {\text{C}}_{{{11}}} + {\text{2C}}_{{{12}}} $$

Combining these two equations and eliminating C_11_, one obtains

3 $${C}_{12}-{C}_{44}=B\left(1-\frac{5{G}_{v}}{3B}\right)$$

From Eq. (3), Pettifor’s condition for insipient brittleness, (C_12_ − C_44_) < 0, occurs when G_v_/B > 0.6. It is necessary to point out that the Voigt shear modulus represents the upper bound of the macroscopic shear modulus G, which value is between Voigt and Reuss estimates and is often taken as an arithmetic average of these two values^11^. The Reuss shear modulus G_r_ is the lower bound for G and is defined as

4 $${G}_{r}=\frac{5{C}_{44}({C}_{11}-{C}_{12})}{4{C}_{44}+3({C}_{11}-{C}_{12})}=\frac{{C}_{44}(5{G}_{v}-3{C}_{44})}{3{G}_{v}-{C}_{44}}$$

while the Reuss bulk modulus B_r_ is equal to Voigt bulk modulus B_v_ and is given by Eq. (2), i.e. B_r_ = B_v_ = B^11^. For isotropic cubic crystals, (C_11_ − C_12_)/2 = C_44_ and G = G_r_ = G_v_ = C_44_. Therefore, Pettifor’s condition for insipient brittleness of isotropic cubic crystals occurs when G/B > 0.6.

For anisotropic cubic crystals, (C_11_ − C_12_)/2 ≠ C_44_, G_r_ < G < G_v_, and the ductile-to-brittle transition occurs at a G/B ratio smaller than 0.6. Indeed, in this case G_r_ and G = (G_v_ + G_r_)/2 can be expressed as functions of G_v_ and the Zener anisotropy ratio A = (2C_44_)/(C_11_ − C_12_):

5a $${G}_{r}=\frac{25A}{(2+3A)(3+2A)}{G}_{v}$$

5b $$G=\frac{3{A}^{2}+19A+3}{(2+3A)(3+2A)}{G}_{v}$$

When A = 2, G_r_ = 0.89G_v_, G = 0.95G_v_ and the ductile to brittle transition occurs at G/B = 0.57. This G/B value is typically used for the Pugh criterion^5^. For materials with A = 3 (e.g. Ag, Au and Cu all have A ≈ 3), G_r_ = 0.76G_v_ and G = 0.88G_v_, and, according to Eq. (3), these materials are brittle if G/B > 0.53. Our analysis thus gives physical meaning to the Pugh criterion. Indeed, for an isotropic cubic crystal, G = G_v_ and Pettifor’s condition for insipient brittleness, (C_12_ − C_44_) < 0, occurs when G/B > (G/B)_dbt_ = 0.6. (The subscript ‘dbt’ indicates that the value corresponds to the ductile to brittle transition boundary.) Because these two conditions occur simultaneously, this indicates, that an increase in G/B ratio above 0.6 should result in the formation of directional, covalent bonding and brittleness, in accord to Pettifor’s theory^8,9^. Additionally, our analysis also shows that in anisotropic cubic crystals the transition from non-directional, metallic bonds to directional, covalent bonds occurs at a smaller G/B ratio, as increasing A beyond unity decreases the critical G/B value.

Equations (3) and (5) indicate that a linear relationship between C”/B and G/B should be met, with a negative slope increasing with increasing A:

6 $$\frac{C"}{B}=1-\frac{5(2A+3)(2+3A)}{3(3{A}^{2}+19A+3)}\frac{G}{B}$$

Accordingly, Pettifor’s brittleness condition C” < 0 becomes equivalent to the Pugh condition when modified to account for elastic anisotropy:

7 $$\frac{G}{B}<\frac{3(3{A}^{2}+19A+3)}{5(2A+3)(2+3A)}$$

**Figure 2:**
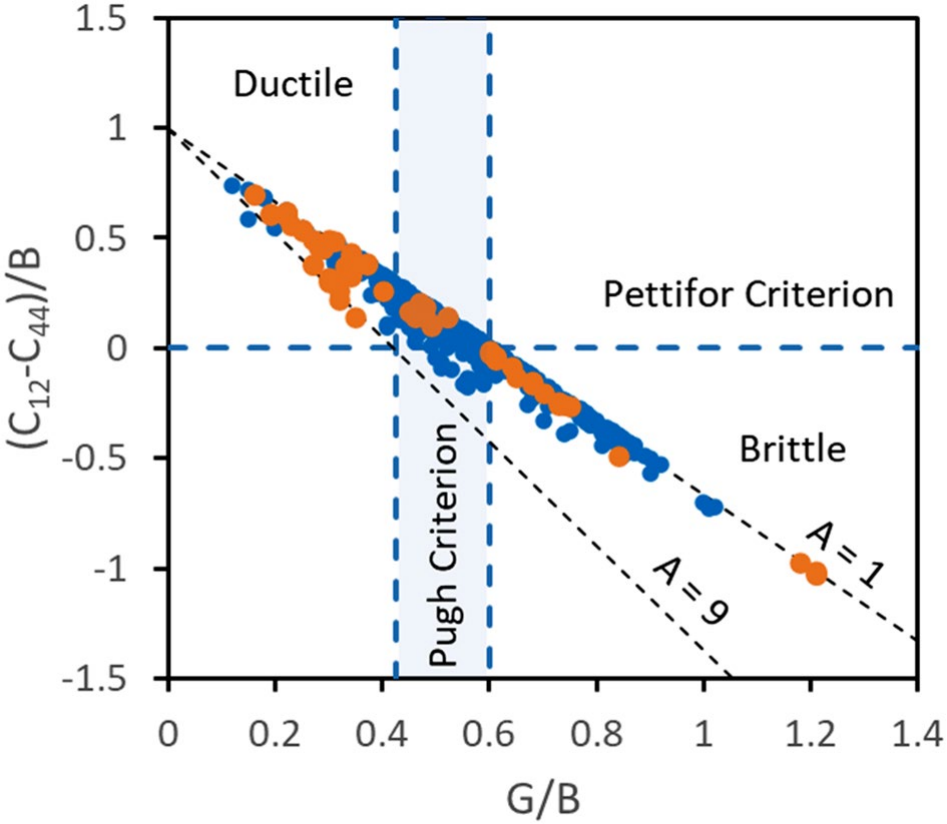
Correlation between C”/B = (C_12_–C_44_)/B and G/B for 308 compounds (blue points) and 24 metals (brown points) with cubic crystal structures analyzed in^5^. The linear trendlines are described by Eq. (6) for isotropic (A = 1) and highly anisotropic (A = 9) materials. The vertical band corresponds to G/B in the range of 0.42 to 0.6 to account for the effect of elastic anisotropy on the value of G at C_12_–C_44_ = 0.

To verify this, we plotted the experimental data used by Niu et al*.*^5^ in the coordinates of C”/B versus G/B in Fig. 2. It is found that indeed the data follow a linear relationship between these two parameters. The spread of the data is due to different A values, which vary from 0.14 to 9 for the analyzed materials. To visualize the effect of elastic anisotropy, two trendlines (Eq. 6) are plotted, one for A = 1 and another for A = 9 (or A = 1/9, as Eq. (6) provides equivalent solutions for A = A’ and A = 1/A’). It is seen that the dependence of C”/B on G/B becomes stronger and Pettifor’s condition for incipient brittleness occurs at smaller G/B values with increasing elastic anisotropy. The Pettifor criterion gives a discrete boundary between intrinsically ductile and brittle materials at (C_11_ − C_12_)/B = 0, but the present analysis shows that this translates to a range in G/B to delineate brittle from ductile behavior (Eq. 7). For elastically isotropic materials this transition occurs at (G/B)_dbt_ = 0.6, but the critical G/B value decreases to (G/B)_dbt_ = 0.42 with increasing the elastic anisotropy (see Fig. 2). This distributed boundary is fully consistent with Pugh’s original analysis, where a specific value in G/B was not proposed^2^.

Our analysis also shows that the bulk modulus rather than Young’s modulus should be used to normalize the Cauchy pressure and obtain the linear relationship between the bulk modulus reduced Cauchy pressure and shear modulus. On the other hand, considering that $E=\frac{9BG}{3B+G}$ and using Eq. (3) one can show that C”/E is a non-linear function of G/B:

8 $$\frac{C"}{E}=\frac{B}{9{G}_{v}}\left(3+\frac{{G}_{v}}{B}\right)\left(1-\frac{5{G}_{v}}{3B}\right)$$

This explains the “hyperbolical” correlation between C”/E and G/B previously found by Niu et al*.*^5^ (see yellow dashed line in Fig. 1b).

It is necessary to point out, that the above analysis was conducted for materials with cubic crystal structures and the results may not be applied to materials with non-cubic crystal structures. Reduced symmetry in non-cubic crystal structures requires expansion of the C_ij_ matix to include terms beyond just C_11_, C_12_ and C_44_, dramatically complicating relationships between C_ij_, G and B. A universal elastic anisotropy index that includes non-cubic crystals, A^U^, has been derived from variational principles of elasticity and consideration of the spherical and deviatoric components of the elastic constants^13^. While this opens the possibility of a universal ductility criterion based on elastic properties alone, quantifying A^U^ requires knowledge of the fourth order deviatoric components of the elasticity tensor, making its general application impractical.

In conclusion, our analysis shows that two classical brittle-to-ductile transition criteria, Pugh’s modulus ratio and Pettifor’s Cauchy pressure, are equivalent for materials with cubic crystal structures and they should be considered as one, the Pugh-Pettifor criterion. We derive a new equation establishing the equality between these previous two criteria.

## Methods

Analytical methods were used to derive the relationships, Eqs. (3) through (8). These relationships were verified using the experimental data from Ref.^5^.
